# RBD_206_-sc-dimer induced robust cross-neutralization against SARS-CoV-2 and variants of concern

**DOI:** 10.1038/s41392-021-00798-8

**Published:** 2021-11-10

**Authors:** Chuge Zhou, Xiaodong Zai, Ziqing Zhou, Ruihua Li, Yue Zhang, Yaohui Li, Ying Yin, Jun Zhang, Junjie Xu, Wei Chen

**Affiliations:** Beijing Institute of Biotechnology, No. 20 Dongdajie Street, Fengtai District, 100071 Beijing, China

**Keywords:** Vaccines, Vaccines

**Dear Editor**,

To date, the severe acute respiratory syndrome coronavirus 2 (SARS-CoV-2) has caused more than 223 million confirmed cases of coronavirus disease 2019 (COVID-19), including 4.6 million deaths (https://covid19.who.int/). Since 2020, several SARS-CoV-2 variants including Alpha (B.1.1.7), Beta (B.1.351), Gamma (P.1), and Delta (B.1.617.2) with immune evasiveness have emerged and fast spread. In a global research effort, scientists proposed multiple effective vaccine strategies to respond to the ongoing COVID-19 pandemic. Most vaccines currently in use or in clinical development target the SARS-CoV-2 Spike (S) glycoprotein, a homotrimer decorates on the viral surface; within it, a distinct receptor-binding domain (RBD, residues 331–524) is responsible for mediating cell entry and interaction with host receptor angiotensin-converting enzyme 2 (ACE2).

Study also shown RBD-directed neutralizing antibodies(nAbs) are less-compromised by SARS-CoV-2 mutations due to their diverse RBD binding modes,^1^ therefore it could better protect against circulating variants. To ameliorate the limited immunogenicity of RBD, in early studies on SARS, N-glycan sites on RBD have been found to be promising modification targets.^2^ Two N-glycan sites (N331, N343) confirmed lies in the SARS-CoV-2 RBD, which likely play a role in protein folding and immune evasion.^3^ Previous studies have also demonstrated a universal dimeric form of CoV RBD (RBD single-chain dimer) that contains two tandem full-length RBD subunits (R319-K537) and boosts immunogenicity in mice.^4^ Here, we present the RBD_206_ (I332-K537)-dimer, a glycan-truncated immunogen combining glycosylation modification and structure-guided design. It has been shown to be a remarkable immunogen form that generates more antibodies, higher neutralizing activity and sufficient cross-reactive neutralization against SARS-CoV-2 wild-type, B.1.351 (Beta) variant and B.1.617.2 (Delta) variant.

RBD_206_, based on RBD_219_ (R319-K537) of wild-type SARS-CoV-2, sequenced from R319 to N331 was deleted, as shown in Fig. 1a. RBD_219_, RBD_206_, RBD_219_-dimer, and RBD_206_-dimer were expressed in Expi293F cells. The recombinant proteins with different molecular wight were verified by sodium dodecyl sulphate–polyacrylamide gel electrophoresis Coomassie-stained gels (Supplementary Fig. S1b). We then analyzed the glycosylation of RBD dimers, The truncated RBD_206_ monomer and dimers shown less PNGase F activity (Fig. 1b). The glycopeptides generated by trypsin and chymotrypsin were analyzed by liquid chromatography mass spectrometry, and the N-linked/O-linked glycosylation siteswere determined (Fig. 1b). Subsequently, the BIAcore assay demonstrated that RBD_206_ was bound to hACE2 receptor with similar affinity (1.16 nM) as RBD_219_ monomer (1.52 nM) (Supplementary Fig. S2a, b), while two corresponding dimeric RBDs (2.30 × 10^−2^ nM, 4.77 × 10^−2^ nM) showed higher receptor affinity (Supplementary Fig. S2c, d), suggesting the exposure of two RBMs may account for improved binding affinities. CD spectrum further indicated the similarities in the structure between two monomers and single-chain dimeric RBDs (Supplementary Fig. S1c).

**Fig. 1:**
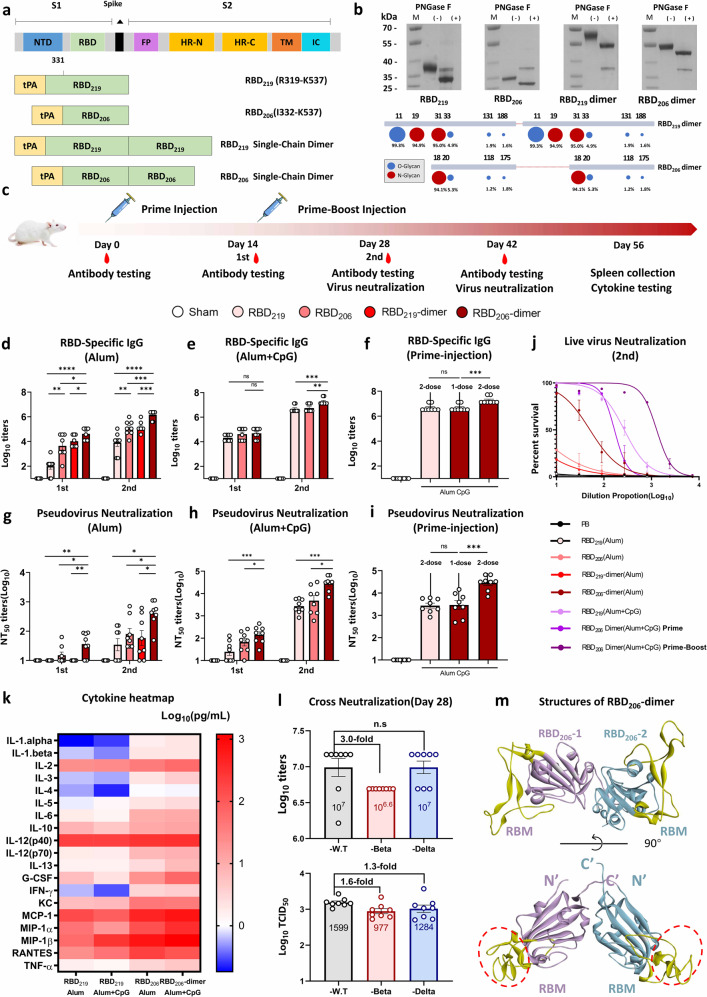
SARS-CoV-2 RBD_206_-sc-dimer vaccine elicited a robust cross-reactive neutralizing response in mice. **a** Expression profiles of SARS-CoV-2 RBD proteins. Wild-type RBD_219_ monomer (R319-K537), RBD_206_ (I332-K537),^2^ RBD_219_-dimer,^4^ and RBD_206_-dimer were expressed in Expi293F. **b** The tPA-tagged proteins were harvested from supernatant and then purified, as verified by gel electrophoresis and HPLC, see also Supplementary Fig. S1a. RBD-based proteins were identified under reducing and unreducing conditions (Supplementary Fig. S1b). Five micrograms of of SARS-CoV-2 RBD-based antigens before and after PNGase-F treatment were loaded on a 4–12% Tris-glycine gel in a reduced condition. The size reduced after PNGase-F treatment suggested that RBD_219_ was N-glycosylated while RBD_206_ was less N-glycosylated. The graph below summarized quantitative mass spectrometric analysis of the glycan population present at individual N-linked/O-linked glycosylation sites, principal glycan types were simplified into two colors, O-linked glycan series were colored blue, N-linked glycans were red, and the circular shapes summarized the relative intensities of these glycans. **c** Schematic diagram of immunization and serum sample collection. In the pilot study, BalB/c mice (6–8 weeks, *n* = 8) were immunized at day 0 and day 14 with 5-µg doses of RBD_219_/RBD_206_-monomer and RBD_219_/RBD_206_-dimer with 50-µg aluminum hydroxide (alum). PB was administered as control group. In the following study, BalB/c mice (6–8 weeks, *n* = 8) were immunized with antigen in the combination of alum and CpG2006 (25-µg) following the same procedure. Serum samples were collected at days 14, 28, and 42 as indicated. Spleen samples were collected at day 56. **d**–**f** RBD-specific IgG titers were tested by ELISA. Naive mice (BalB/c, *n* = 8 per group) were immunized with either RBD_219_, RBD_206_ or RBD_219_ dimer, or with RBD_206_ dimer adjuvanted with alum as shown in **d**. BalB/c mice (*n* = 8 per group) were immunized with either RBD_219_, RBD_206_ or with RBD_206_ dimer in the joint of alum and CpG as shown in **e**. In **f**, the group received prime-injection of RBD_206_ dimer with alum and CpG was highlighted. Data represented antibody titers on Day 28 post prime-injection. The experiments were further repeated twice, and similar results were obtained. All the data were presented as mean ± SEM. ***P < 0.001; **P < 0.01; *P < 0.1. ns: No significant difference. **g**–**i** Pseudovirus-based neutralization assays were performed to detect neutralizing antibody (NAb) titer against SARS-CoV-2, Neutralizing antibodies of wild type SARS-CoV-2 pseudoviruses were assessed in 293T-ACE2 cells (*n* = 8), and the neutralizing level was shown as 50% neutralizing titer (NT_50_) (*n* = 8). Naive mice (BalB/c, *n* = 8 per group) were immunized with either RBD_219_, RBD_206_ or RBD_219_ dimer, or with RBD_206_ dimer adjuvanted with alum as shown in **g**. BalB/c (*n* = 8 per group) were immunized with either RBD_219_, RBD_206_ or with RBD_206_ dimer adjuvanted with alum and CpG as shown in **f**. In **i** the group received prime-injection of RBD_206_ dimer with alum and CpG was highlighted. Data represent neutralizing antibody titer at day 28 after prime-injection. The experiments were further repeated twice, and similar results were obtained. All the data were presented as mean ± SEM. ***P < 0.001; **P < 0.01, *P < 0.1. ns: No significant difference. **j** Neutralization titer of sera collected at day 28 against live SARS-CoV-2 were shown as serial dilution curves (*n* = 8). All the data were presented as mean ± SEM. The experiments were performed in duplicate, and similar results were obtained. **k** The level of cytokines secreted by stimulated splenocytes of mice (*n* = 4) vaccinated was shown by Log_10_Concentration (pg/ml) in the heat map. The RBD peptide pool was used as a mock antigen to investigate the effect of splenocytes. In all, 2 µg/ml peptide mixture was then co-incubated with splenocytes for at least 48 h at 37 °C. Both stimulated and unstimulated splenocyte supernatants were collected, and cytokines in the supernatants were detected by ELISA using Bio-Plex Pro Mouse Cytokine Crp I Panel 19-plex. **l** Cross-neutralization of serum of immunized BALB/c mice was detected with live wild-type SARS-CoV-2, Beta variant and Delta variant (*n* = 8). Neutralizations at original dilution of 1:30 of serum are shown. Neutralization titers against live wild-type SARS-CoV-2/Beta/Delta variant are shown as individual values (*n* = 8). ***P < 0.001; **P < 0.01, *P < 0.1. ns: No significant difference. **m** RBD_206_ single-chain dimer structures were simulated by Alpha-Fold. Similar to the RBD_219_-sc-dimer,^4^ RBD_206_-sc-dimer contains two tandem repeat domains (I332-K537), otherwise the 13-amino acid–sequence, including N331, has been removed. Two RBD_206_ monomers are colored in violet and pale cyan, respectively. The regions of RBM are represented in light yellow ellipses

In order to evaluate the immunogenicity of the designed RBDs, we assessed IgG titers of BalB/c mice (*n* = 8) immunized with different antigens and alum according to the two-dose regimen (Fig. 1c). With increased IgG titer against SARS-CoV 2, RBD_206_ was found to be a favorable design (Fig. 1d). Specifically, we found that the RBD-specific binding antibodies in RBD_206_-dimer groups reached ~10^5^ as early as 2 weeks post priming, which indicated that RBD_206_-dimer significantly enhanced the immunogenicity of RBD antigens (Fig. 1d). After boost injection, the IgG titer in RBD_206_-dimer-immunized mice sera increased to ~10^6^ at day 28, which was 13.8-fold higher than that in the RBD_219_-dimer-immunized group. Then neutralizing titer was further tested. After the prime-boost vaccination, neutralizing antibody(nAb) response elicited by the RBD_206_-dimer against pseudovirus was higher than the RBD_219_-dimer with 50% pseudovirus neutralization titer (NT_50_) of ~10^3^ and ~10^2^ respectively (Fig. 1g). As illustrated by the neutralizing curve in Fig. 1j, RBD_206_-dimer maintained its high efficacy in live virus neutralization assay with NT_50_ > 50, while NT_50_ < 30 was elicited by RBD_219_-dimer. We further vaccinated BalB/c mice (*n* = 8) at 0 and 14 days, using 5, 2, and 1 µg of RBD_219_-dimer and RBD_206_-dimer per dose separately, quantified binding Abs to RBD suggested a dose-dependent response to the vaccine. Sufficient binding antibodies were induced by the low-dose RBD_206_-dimer vaccine after prime vaccination (Supplementary Fig. S4).

To further improve the immunogenicity of RBD_206_-dimer, in this study, CpG2006 (Takara), a 24-mer CpG ODN in the toll-like receptor (TLR9) was formulated along with alum as adjuvants. After the two-dose immunization, RBD_219_ induced IgG titer of ~10^6^, while that of ~10^7^ was induced by RBD_206_-dimer (Fig. 1e). In accordance with IgG results, neutralizing antibodies (nAbs) induced by RBD_206_-dimer, strongly inhibited pseudotyped SARS-CoV-2 infection, with NT_50_ up to >10,000 at day 28, which was 10-fold higher than that in the RBD_219_ combined with CpG alum group (Fig. 1h). In the live virus neutralization assay, RBD_206_-dimer with CpG vaccine neutralized over 50% of the live SARS-CoV-2 at the serum dilution ranged from 1:1000 to 1:3000 after two-dose injection, which performing better than other RBD vaccine formulations (Fig. 1j). As expected, formulation with CpG enhanced nAb titers induced by RBD_206_-sc-dimer compared with immunizations with non-formulated antigens.

In the presence of CpG, we then verified immune response induced by prime-injection of RBD_206_-dimer. At 28 days after prime vaccination, IgG titer of ~10^6^ was detected in RBD_206_-dimer single-injection group, which was slightly higher than IgG titer induced RBD_219_-monomer boost vaccination (Fig. 1f). Moreover, the NT_50_ of RBD_206_-dimer vaccine prime-injection group ranged from 1:100 to 1:300, of note, immunization with RBD_219_ formulated with CpG alum elicited similar levels of neutralizing Abs after two-dose injection. Our results indicated that a prime-only vaccination regimen might be enough to elicit sufficient nAbs against SARS-CoV-2.

Given the efficient antibody response, we next quantified vaccine-specific cytokine responses. Consistent with differences observed in IgG subtypes (Fig. S3). We verified a Th1-dominant response as displayed by IgG1 subtype-specific titers (~10^5^) induced by RBD_206_-dimer with alum, which was slightly higher than that induced by RBD_219_. CpG was shown to boost the IgG2a titers. We found that RBD_206_-dimer elicited higher cytokine levels, including IFN-γ+, IL-2+, and TNF-α in RBD peptide-stimulated splenocytes than the RBD_219_ antigen (Fig. 1k). These findings further suggested that RBD_206_-dimer with CpG both induced cellular and boosted humoral immunity.

The emergence of SARS-CoV-2 variants such as Beta and Delta has raised concerns regarding possible reduction in vaccine efficacy. Neutralizing Ab titers are an important correlate of protection against SARS-CoV-2 infection.^5^ We therefore assessed the cross-neutralization of RBD_206_-dimer against Beta and Delta variants. Here, we found that nAbs elicited by RBD_206_-dimer formulated with CpG alum could potently neutralize wild type Beta as well as Delta variant. We observed that the Beta variant isolated was 1.6-fold less sensitive to the sera compared with the wild type, and the Delta strain was 1.3-fold loss of neutralization sensitivity compared with the reference wild type (Fig. 1l), which was in accordance with anti-RBD-binding antibodies. The marginal change of nAb titer provides supportive evidence that RBD_206_-dimer vaccines could induce RBD-targeted nAbs, maintaing high resistance to SARS-CoV-2 mutations.

At present, multiple RBD-based antigens including RBD-monomer, RBD-dimer, and RBD-trimer, etc. are being developed into diverse recombinant subunit vaccines. In this report, we proposed RBD_206_-dimer subunit vaccine. Further neutralizing assays showed that RBD_206_-dimer created about a 10-fold increase in both binding and neutralizing Abs against wild-type SARS-CoV-2 than RBD_219_-dimer. As shown in Alpha-Fold simulation of protein structure (Fig. 1m), the deletion of N331-glycan was likely to reduce the glycan masking, and increase the exposure area of antigen binding sites, thereby enhancing the neutralizing antibodies response.^4^ Additionally, we observed that the immunization of mice with RBD_206_-dimer formulated with CpG alum elicited comparable cross-reactive nAbs against Beta and Delta variants, showing only minimally reduced vaccine effectiveness compared with wild-type SARS-CoV-2. As recent research has suggested, it is likely that RBD-based recombinant proteins could better cope with the immune evasion by inducing diverse RBD-directed nAbs, other than spike-targeted vaccines.^1^

Collectively, our findings highlight RBD_206_ single-chain dimeric repeats, as a promising candidate vaccine against COVID-19, which may improve vaccine efficacy against circulating SARS-CoV-2 variants.

## Supplementary information

Supplementary Materials

## Data Availability

The data are available from the corresponding author on reasonable request.
