# Supplementary material for: RBD206-sc-dimer induced robust cross-neutralization against SARS-CoV-2 and variants of concern
Source: Signal Transduct Target Ther. 2021 Nov 10;6:390. doi: 10.1038/s41392-021-00798-8 (PMC8578908; doi:10.1038/s41392-021-00798-8)
Supplement: Supplementary file 1 — Supplementary Materials [file 41392_2021_798_MOESM1_ESM.docx]

Supplementary Materials for

**RBD_206_-sc-Dimer Induced Robust Cross-neutralization against SARS-CoV-2 and Variants of Concern**

Chuge Zhou^§^, Xiaodong Zai^§^, Ziqing Zhou, Ruihua Li, Yue Zhang, Yaohui Li, Ying Yin, Jun Zhang, Junjie Xu*, Wei Chen*

1 Beijing Institute of Biotechnology, No. 20 Dongdajie Street, Fengtai District, Beijing 100071, China.

§These authors contributed equally to this work.

*Correspondence to:

Wei Chen, Beijing Institute of Biotechnology

Beijing, China, E-mail: cw0226@foxmail.com (W.C)

Junjie Xu, Beijing Institute of Biotechnology

Beijing, China, E-mail: xujunjie@sina.com (J.X)

**This PDF file includes:**

[Methods and materials](#_Toc82945042)

[Figure S1](#_Toc82945043)

[Figure S2](#_Toc82945044)

[Figure S3](#_Toc82945045)

[Figure S4](#_Toc82945046)

# Methods and materials

**1. Cell lines and cell culture**

293T cells that highly express ACE2 receptor (ACE2-293T) were generated by stably transfecting human angiotensin–converting enzyme 2 (ACE2) gene into HEK293T cells under the pressure of Puromycin in the lab^1^. All the cells were cultured in Dulbecco’s modified Eagle’s medium (DMEM, Thermo Scientific, USA), containing 10% fetal bovine serum (FBS, Thermo Scientific, USA), penicillin (100 units/mL) and streptomycin (100 μg/mL) at 37°C in 5% CO_2_ incubator.

**2. Construction and purification of RBD-based antigens**

Wild-type RBD_219_ monomer and RBD_219_-dimer, proteins encoding the full-length spike-RBD of the wild-type Wuhan-Hu-1 strain (NC_045512.2) was constructed as stated previously^1^. The wild-type RBD gene (R319-K537) was codon-optimized for mammalian cell expression and synthesized.

RBD_219_-sc-dimer of SARS-CoV-2 was two RBD (S protein R319-K537) connected as tandem repeat. For RBD_206_-monomer, the codon-optimized RBD_219_ gene of wild-type strain with the glycan cleavage site mutation (R319-N331 deletion) was synthesized and cloned into the plasmid. RBD_206_-sc-dimer of SARS-CoV-2 was two RBD_206_ (S protein I332-K537) connected as tandem repeat. For each construct, the tissue plasminogen activator (tPA) signal peptide (1–23 aa) and a hexa-His tag were added to the protein N terminus for protein secretion and further purification processes respectively.

After sequencing identification, the plasmids with the target gene were co-transfected into Expi293F mammalian cell according to the manufacturer’s instructions. The transfected cells were cultured on a shaker under the following conditions: 120 rpm, 37°C, relative humidity ≥ 80%, and carbon dioxide concentration of 8%. After 72 h of transfection, the cell culture medium was centrifuged at 3000 g for 15 min. The filtered supernatant was collected for subsequent purification. The target RBD proteins were purified by a HisTrap affinity chromatography column (GE Healthcare). The concentrations of the target proteins were measured by ultraviolet spectrophotometer analysis following the manufacturer’s instructions.

The purified proteins were stored in 20 mM PB, pH 7.4 until further use. SARS-CoV-2 RBD_219_，RBD_206_, RBD_219_-dimer and RBD_206_-dimer were treated with PNGase-F (NEB, P0705L) to remove the N-glycans following the vendor’s manual. 5μg of SARS-CoV-2 RBD before and after PNGase-F treatment were loaded on a 4-12% Tris-glycine gel under reduced conditions to evaluate the size. The purity of the SARS-CoV-2 RBD antigens were further evaluated using SE-HPLC (Ultimate® XB-C4, Welch).

**3. Immunization and sampling schedule**

The experiments involving animals were approved by and carried out in accordance with the guidelines of the Institutional Animal Care and Use Committee of Laboratory Animal Center. Specific pathogen–free (SPF) female BALB/c mice aged 6–8 weeks, obtained from Beijing Vital River Laboratory Animal Technologies Co., Ltd. (Beijing, China), were housed and bred in a temperature-, humidity-, and light cycle–controlled animal facility. The mice were randomly divided into multiple groups and were immunized intramuscularly with Primer vaccine (wild-type RBD_219_, N331-glycan truncated RBD (RBD_206_), RBD_219_ single-chain dimer, RBD_206_ single-chain dimer, PB alone as control), with alum adjuvant, or in combination of alum and CpG2006, at day 0, with boost injection at day 14. Sera were collected at weeks 2, 4, and 6 post immunization for RBD-specific IgG ELISA assays, pseudovirus neutralization assays and live SARS-CoV-2 microneutralization assays.

**4. ELISA**

RBD-specific serum antibodies (including IgG, IgG1 and IgG2a) were determined by enzyme-linked immunosorbent assay (ELISA). ELISA assays were obtained from ACRO Biosystems (Beijing, China) and Sino Biological, Inc. (Beijing, China). Ninety-six-well microplates (Corning, USA) with 1 μg/mL RBD proteins coated with carbonate buffer (pH 9.6) were incubated at 4°C overnight. The plates were then blocked at 37°C for 1 h with PBS (pH 7.4) in 2% bovine serum albumin (BSA, Sigma, USAS). After washing with PBS with 0.2% (v/v) Tween 20 (PBST) 3 times, serial dilutions of sera were added to the plates and incubated at 37°C, after 1-h incubation, plates were washed by PBST three times, HRP-conjugated goat anti-mouse IgG (Abcam, UK, 1:10,000 dilution) was added to the plates, and the plates were incubated at 37°C for 1 h and washed with PBST three times. The assay was developed for 6 min at RT with 100 μL of TMB substrate solution (Solarbio, China), stopped by adding of 50 μL of stop solution (Solarbio, China) and then measured at 450 nm/630 nm (SPECTRA MAX 190, Molecular Device, USA). Endpoint titers were calculated as the dilution that exceed 2.1-folded value of the background.

**5. Generation of pseudovirus and neutralization assay**

The SARS-CoV-2 pseudovirus system was generated by our team in a previous study^1^. Briefly, the spikes sequences of SARS-CoV-2 wild-type Wuhan-Hu-1 strain (NC_045512.2) were synthesized and subcloned into pCAGGS plasmid (Youbio, China). A total of 7 × 10^6^ HEK293T cells were seeded into a 10-cm plate and co-transfected with 23 μg of pNL4-3.Luc-R-E− and 1 μg of pCAGGS-spike by the TurboFect Transfection Reagent (Thermo Scientific, USA). The supernatants containing pseudovirus were collected, filtered, aliquoted and frozen at −80°C after 48 h. Serum was heated at 56°C for 30 min, Serial dilutions of inactivated sera were mixed with the titrated pseudovirus, incubated for 1 h at 37°C in 5% CO2 incubator and added to ACE2-293T cells in 96-well microplate. Cells were lysed 48 h later, and luminescence was measured using BriteLite PLUS Kit (PerkinElmer). The EC50 neutralization titers was determined as the dilution for which luciferase activity reached half of that of the negative control using the Reed-Muench method. The initial dilution of sera (1:30) was set as the limit of confidence of the assay.

**6. Live SARS-CoV-2 microneutralization assay**

The neutralizing activity of sera from the mice against live SARS-CoV-2 wild-type (Wuhan) , Beta^2^ and Delta lineage^3^ were assessed using a microneutralization assay^1^. Serial dilutions of sera were incubated with 100 TCID50 of SARS-CoV-2- wild-type (Wuhan), Beta and Delta lineage at 37°C for 1 h. Sera-virus complexes were added to pre-plated Vero E6 cell monolayers in 96-well plates and incubated for 48–72 h. The cells were stained with 0.05% crystal violet for 40 min. The OD was measured at 570 nm/630 nm after the addition of the decolorization solution. Neutralization results were analyzed using the Reed-Muench method to estimate the dilution of sera required for half-maximal neutralization of infection (NT50 titer). The initial dilution of sera (1:10) was set as the limit of confidence of the assay.

**7. Cytokine measurement by Luminex chip detection**

For cytokine measurements, Luminex chip detection was performed by Wayen Biotechnologies (Shanghai, China). In brief, mouse splenocytes were collected at day 56 after two-dose injection. Splenocytes treated with or without RBD peptides pool were maintained in 10% FBS Roswell Park Memorial Institute (RPMI) 1640 culture, and incubated in 96-well plates for 48 h at 37°C. After centrifuge, supernatants were processed with the Bio-Plex Mouse Cytokine 19-Plex. The Bio-Plex Pro Mouse Cytokine Crp I Panel 19-plex was used in accordance with the manufacturer’s instructions. Array analysis was performed using the Bio-Plex Protein Array system (Bio-Rad Laboratories, USA).

**8. Statistical analysis**

GraphPad v.7.0 was used for statistical analysis. Continuous data were presented as the mean ± SEM, and antibody titer data were presented as log_10_ transformed before analysis. Paired t-test and one-way ANOVA with Tukey’s multiple comparisons tests were applied for the significant difference analysis where applicable. Statistically significant differences were considered (*P < 0.05; **P < 0.01; ***P < 0.001; ****P < 0.0001). Error bars throughout all figures represent mean ± SEM.

# Figure S1





**Figure S1. Representative characterization of SARS-CoV-2 RBD_219_/RBD_206_ -monomer and RBD_219_/RBD_206_-sc-dimer protein. (a)** Analysis of RBD-based proteins by high-performance liquid chromatography (HPLC) (**b)** SDS–polyacrylamide gel electrophoresis analysis of the SARS-CoV-2 RBD-based antigens(under reducing/non-reducing conditions) expressed in Expi293F mammalian cell. **(c)** The secondary structure of SARS-CoV-2 RBD_219_/RBD_206_ -monomer and RBD_219_/RBD_206_-sc-dimer were analyzed by collecting circular dichroism (CD) spectrum graphs in the far UV region (180–260 nm).

# Figure S2

#
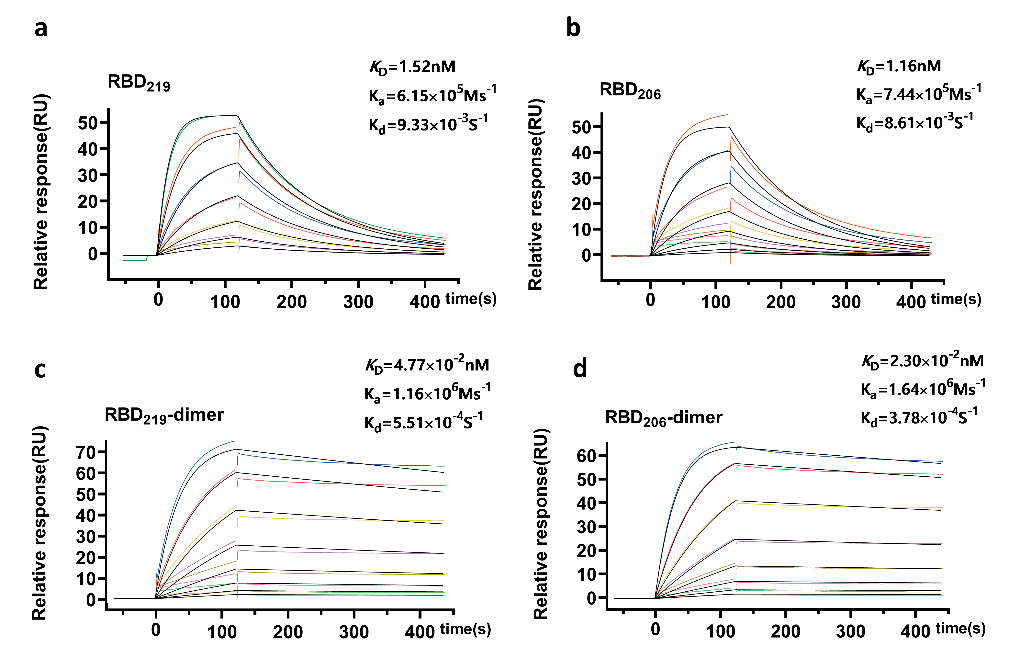


**Figure S2. BIAcore diagrams of SARS-CoV-2 RBD_219_/RBD_206_-monomer and RBD_219_/RBD_206_-sc-dimer bound to hACE2 receptor** Binding of S-RBD to ACE2-ECD (a) was measured by experiments in the Biacore 8K (GE Healthcare) at 25°C. The KD value of the four RBD mutants (RBD_219_, RBD_206_, RBD_219_-dimer, and RBD_206_-dimer) binding affinity for ACE2 was calculated from all the binding curves based on their global fit to a 1:1 binding model. The *K*D values of wt RBD_219_, RBD_206_, RBD_219_-dimer, and RBD_206_-dimer binding affinity for ACE2 was calculated and analyzed using BIAevaluation 4.1 (GE Healthcare).

**Figure S3**

**Figure S3. RBD-Specific IgG subtype**. Sera from mice after two-dose injection with RBD_219_ monomer or RBD_206_-sc-dimer were analyzed for the IgG subclass specificity of their antibody response by ELISA (n=8). Data were expressed as the mean ± SEM. P-values < 0.05 were considered statistically significant (*P < 0.05; **P < 0.01; ***P < 0.001; ****P < 0.0001).

# Figure S4

**Figure.S4**. **Dose-dependent response of RBD-Specific IgG**. BalB/c mice (6–8 weeks, n=8) were immunized at day 0 and day 14 (n=8) intramuscularly with a range of doses of RBD-antigens (5 µg, 2µg and 1 µg) with 50-µg aluminum hydroxide (alum)according to prime-boost regimen. Serum samples were collected 14, 28 days post prime injection as indicated. RBD-specific IgG titers were tested by ELISA. Data were expressed as the mean ± SEM. P-values < 0.05 were considered statistically significant (*P < 0.05; **P < 0.01; ***P < 0.001; ****P < 0.0001).

**REFERENCES**

1. Wu S, et al. A single dose of an adenovirus-vectored vaccine provides protection against SARS-CoV-2 challenge. *Nat Commun*. 2020;11(1):4081. doi:10.1038/s41467-020-17972-1

2. Tegally H, et al. Detection of a SARS-CoV-2 variant of concern in South Africa. *Nature*. 2021;592(7854):438-443. doi:10.1038/s41586-021-03402-9

3. Ferreira IATM,, et al. SARS-CoV-2 B.1.617 Mutations L452R and E484Q Are Not Synergistic for Antibody Evasion. *J Infect Dis*. 2021;224(6):989-994. doi:10.1093/infdis/jiab368
